# Enterovirus A71 utilizes host cell lipid β-oxidation to promote its replication

**DOI:** 10.3389/fmicb.2022.961942

**Published:** 2022-09-28

**Authors:** Xiuwen Yang, Jiayi Chen, Zixin Lu, Shan Huang, Shihao Zhang, Jintai Cai, Yezhen Zhou, Guanhua Cao, Jianhai Yu, Zhiran Qin, Wei Zhao, Bao Zhang, Li Zhu

**Affiliations:** ^1^BSL-3 Laboratory, Guangdong Provincial Key Laboratory of Tropical Disease Research, School of Public Health, Southern Medical University, Guangzhou, China; ^2^Department of Epidemiology, School of Public Health, Southern Medical University, Guangzhou, China

**Keywords:** Enterovirus A71, replication, endoplasmic reticulum stress, lipid metabolism, β-Oxidition

## Abstract

Enterovirus A71 (EV-A71) is a major pathogen that causes severe and fatal cases of hand-foot-and-mouth disease (HFMD), which is an infectious disease that endangers children’s health. However, the pathogenic mechanisms underlying these severe clinical and pathological features remain incompletely understood. Metabolism and stress are known to play critical roles in multiple stages of the replication of viruses. Lipid metabolism and ER stress is an important characterization post viral infection. EV-A71 infection alters the perturbations of intracellular lipid homeostasis and induces ER stress. The characterizations induced by viral infections are essential for optimal virus replication and may be potential antiviral targets. In this study, we found that the addition of the chemical drug of ER stress, PKR IN, an inhibitor, or Tunicamycin, an activator, could significantly reduce viral replication with the decrease of lipid. The replication of viruses was reduced by Chemical reagent TOFA, an inhibitor of acetyl-CoA carboxylase (ACC) or C75, an inhibitor of fatty acid synthase (FASN), while enhanced by oleic acid (OA), which is a kind of exogenous supplement of triacylglycerol. The pharmacochemical reagent of carnitine palmitoyltransferase 1 (CPT1) called Etomoxir could knock down CPT1 to induce EV-A71 replication to decrease. This suggests that lipid, rather than ER stress, is the main factor affecting EV-A71 replication. In conclusion, this study revealed that it is the β-oxidation of lipid that plays a core role, not ER stress, which is only a concomitant change without restrictive effect, on virus replication.

## Introduction

Hand, foot and mouth disease (HFMD) is an infectious disease that mainly infects the healthy of children under the age of 5 years. Enterovirus A71 (EV-A71) is one of the main pathogens that cause HFMD. The HFMD caused by EV-A71 may develop into severe neurological disease with complications such as myocarditis, pulmonary edema and aseptic meningitis. They have a rapid development and a high fatality rate (Wang et al., 1999). However, the pathogenic mechanisms underlying these clinical and pathological features are not fully understood. Metabolism and stress have played a key role in multiple stages of virus replication (Zhang et al., 2019; Thyrsted and Holm, 2021). Lipid metabolism and ER stress is an important characterization post viral infection.

Lipid metabolism is ubiquitous in various cells and plays an important role in the normal growth, development and differentiation of cells. Many studies have shown that some viruses can use intracellular lipid metabolism to promote their own replication after infecting cells (Randall, 2018; Abu-Farha et al., 2020; Zhao et al., 2021). The *de novo* fatty acid biosynthetic pathway produces the long-chain fatty acids palmitate, triglycerides, and lipid droplets. Triglycerides in lipid droplets are utilized for several key biological functions such as phospholipid synthesis, energy production, biofilm synthesis and cell signaling (Liu et al., 2010). Several different viruses, such as HCMV (Spencer et al., 2011), dengue virus (DV) (Heaton et al., 2010), and hepatitis C virus (HCV) (Yang et al., 2008) can manipulate *de novo* fatty acid biosynthesis during infection. The lipid metabolism is closely related to ER (Zhou and Liu, 2014; Han and Kaufman, 2016). Lipid metabolism disorder (Han and Kaufman, 2016; Zhao et al., 2020), external oxidative stress (Ochoa et al., 2018), viral infection (Mehrbod et al., 2019; Shaban et al., 2021) and other changes can cause ER stress. ER stress is a self-protection mechanism of cells. When cells are stimulated by various external factors, cellular homeostasis is disrupted, ER stress is generated and unfolded protein response (UPR) is triggered to help clear the unfolded and misfolded proteins, thereby restoring ER homeostasis (Rashid et al., 2015).

Studies have shown that EV-A71 infection can cause perturbation of lipid homeostasis (Yan et al., 2019) and ER stress (Jheng et al., 2010, 2012, 2016). But little research has been done on the roles of lipid metabolism and ER stress in EV-A71 replication.

## Materials and methods

### Antibodies and reagents

The following antibodies and reagents were used: mouse anti-β-actin and mouse anti-BiP (Proteintech, United States); rabbit anti-EV-A71 VP1(Genetex, United States); rabbit anti-CPT1 and rabbit anti-PLIN2(Abcam, China); Cell Counting Kit-8 (CCK8, Dojindo, China); PKR-IN-C16, Tunicamycin and Etomoxir (MedChemExpress, United States); Oleic acid (OA), and 5-tetradecyloxy-2-furoic acid (TOFA; Sigma- Aldrich, United States); C75 (Selleck, China); nontargeting siRNA (SiNC) and targeting CPT1 (SiCPT1; RiboBio, China); Lipofectamine 3000 reagent (Invitrogen, United States).

### Cell culture, virus isolation, and titer determination

Human umbilical vein endothelial Cells (HUVECs) and Human rhabdomyosarcoma (RD) were cultured in Dulbecco’s modified Eagle’s medium (DMEM, Gibco, United States) containing 10% fetal bovine serum (FBS; Gibco, United States) at 37°C in 5% CO2. EV-A71 virus is preserved in this laboratory. RD cells were infected with viruses, and the culture supernatant was harvested according to the cytopathic effect (CPE). Viruses were stored at −80°C, and viral titers were determined in RD cells by measuring the median tissue culture infectious dose (TCID50).

### siRNA transfection

Cells were plated in a 12-well plate (5 × 10^4^ cells/well). The next day, siRNA was transfected into the cells using Lipofectamine 3,000 and DMEM. HUVECs were infected with EV-A71 after siRNA transfection for 24 h. The sequence of the siRNA specific for CPT1 was 5′-CCAUGAAGCUCUUAGACAATT −3′.

### Cell viability assay

HUVECs (10^4^ cells/well) were plated in a 96-well plate, processed in different ways (treated with chemical drugs or transfected with siRNA for 24 h) and then infected with EV-A71 for 12 h. Then, 0.1 volume of CCK-8 solution was added to each well and incubated for 4 h at 37°C. The absorbance of each well at 450 nm was measured using a microplate reader (Infinite M200, Tecan, Switzerland). The viability of cells without any treatment was set as 100%, and the viability of the other groups was calculated.

### Fluorescence-based quantitative PCR

Total RNA at different time points after viral infection or drug treatment was extracted using TRIzol (Accurate Biology, China). Then, 1 μg of total RNA was reverse transcribed to cDNA using Evo M-MLV RT Kit with gDNA Clean (Accurate Biology, China). Real-time quantitative PCR was performed using SYBR^®^ Green Premix Pro Taq HS qPCR Kit (Accurate Biology, China) to detect GAPDH, BiP, ACC, FASN, PLIN2 and CPT1 mRNA or using Bestar qPCR MasterMix (DBI^®^ Bioscience, Ludwigshafen, Germany) to detect EV-A71 mRNA. The specific primers and probes for EV-A71 and the PCR conditions were consistent with the previous method (Zhu et al., 2019). All primers used for RT-qPCR analysis are provided in Table 1. The mRNA expression levels were normalized to those of GAPDH and were expressed as fold changes calculated using the 2^−ΔΔCt^ method.

**Table 1:** Primer table.

Primer	Sequence (5′-3′)
EV-A71-F	CCAGAAGAATTTTACCATGAAGTTGT
EV-A71-R	AGGGCTCTGCTCATACTATC
EV-A71 probe	FAM-CAGACGGGCACTATACAGGGAG-BQ1
CPT1-F	CCCCTCCAGTTGGCTTATCG
CPT1-R	GACATGCAGTTGGCCGTTTC
GAPDH-F	CATCCTGGGCTACACTGAGC
GAPDH-R	AAAGTGGTCGTTGAGGGCAA

### Western blot analysis

After SiCPT transfection 24 h or at various time points after infection, cells were washed twice with 1× phosphate-buffered saline (PBS) and collected into EP tubes by gently scraping them with a cell scraper. Then, cells were lysed with RIPA lysis buffer (Beyotime, China) for 30 min on ice. After centrifuging the cells lysate for 30 min (14,000 rpm at 4°C), the supernatant was left. Protein concentrations were measured with BCA protein assay kit (Bioworld, United States). SDS-PAGE separated proteins were transferred to polyvinylidene fluoride (PVDF) membranes (Bio-Rad, China), which were blocked with 5% BSA in PBST for 2 h at room temperature, and were incubated with the corresponding primary antibody overnight. The next day, membranes were incubated with secondary antibodies (Bioworld, United States) for 1 h at room temperature and proteins were detected with ECL reagents (Bioworld, United States).

### Nile Red analysis

At different time points after viral infection, HUVECs were fixed with 4% polymerize formaldehyde for 10 min. Nile red dye (Sigma, United States) dissolved into 1 mg/ml with PBST was added into the cell sample (1 ml/dish) to dye for 20 min. Nuclei were stained with DAPI (BestBio, Shanghai, China) for 10 min. Images were acquired using LSM 880 confocal microscope (Carl Zeiss, Germany) and were analyzed with ZEN 2.1 Viewer software (blue version).

### TCID50 assay

Supernatants were collected at different time points after infection for viral titer determination. RD cells (10^4^ cells/well) were plated in a 96-well plate. The next day, the cells were incubated with tenfold dilutions of infected-cell supernatants for 1 h, after which the cells were washed with PBS once and cultured in DMEM containing 2% FBS. On day four or five after infection, the number of plates exhibiting the CPE was recorded to determine the viral titer using the Reed–Muench method.

### Transmission electron microscopy assay

HUVECs were pre-treated with drugs and EV-A71-infected with moi 100. Then cells were fixed in 2.5% glutaraldehyde in phosphate buffer (0.01 M, pH = 7.4). After being rinsed with phosphate buffer, the samples were post-fixed in 1% osmium tetroxide for 1 h, rinsed with water, dehydrated in a graded series of ethanol followed by propylene oxide and kept overnight in Epon812 (Sigma-Aldrich, United States). The samples were embedded in Epon812 and were cured in an oven at 60°C. Ultrathin sections were obtained with a Reichert Ultracut E microtome. The sections were stained with uranyl acetate and lead citrate and were observed using a TEM (Hitachi, H-7500, Tokyo, Japan).

### Statistical analysis

Data from three independent experiments were presented as means ± SEMs determined using GraphPad Prism 7. The significance of differences was analyzed by one-way ANOVA in SPSS 24.0. A *p* < 0.05 was considered to indicate a significant difference.

## Result

### EV-A71 infection leads to altered lipid metabolism and ER stress

**Figure 1:**
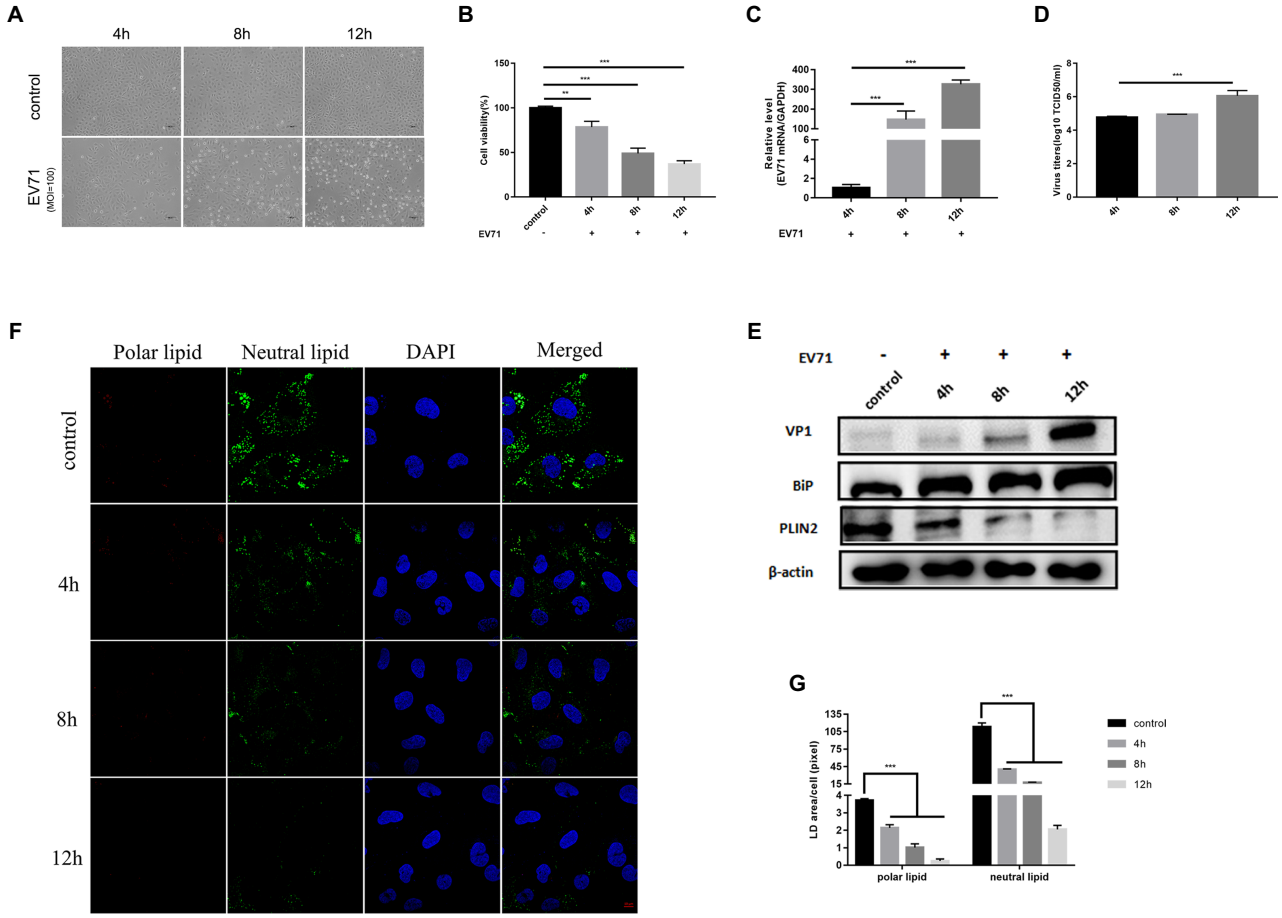
EV-A71 infection of HUVECs caused ER stress and altered lipid metabolism. HUVECs were infected with EV-A71 at moi 100. **(A)** Cytopathic effects were recorded under an inverted light microscope, mock-infected cells were used as negative controls. (scale bar 100 μm). **(B)** CCK8 detected cell viability at 4 h, 8 h and 12 h after infection. **(C)** Intracellular RNA was extracted at 4 h, 8 h and 12 h after infection, the mRNA expression of EV-A71 was calculated with 4 h as control. **(D)** Collects 4 h, 8 h and 12 h after infection in the cell supernatant, the titer of virus was detected by TCID50. The EV-A71 virus titers were 1.5 × 10^7^TCID50/ml, 2.7 × 10^7^TCID50/ml and 5.2 × 10^7^TCID50/ml at 4 h, 8 h and 12 h after infection, respectively. **(E)** The expression levels of VP1, BiP and PLIN2 were detected by western blot at 4 h, 8 h and 12 h after infection. **(F)** Lipid in cells were stained with Nile Red dye at 4 h, 8 h, and 12 h after infection, and the lipid were observed by confocal microscopy. Polar lipid droplets were red and neutral lipid droplets were green. **(G)** The lipid droplet area of each cell was quantitatively calculated. All the experiments were carried out in triplicate. Error bars represent SEM. * means *p* < 0.05, ** means *p* < 0.01, *** means *p* < 0.001.

To investigate the pathogenesis of EV-A71-related manifestations, we first established an *in vitro* model using HUVECs to characterize the changes induced by EV-A71 infection. Compared to cancer cells with abnormal baseline metabolism, HUVECs represent human primary cells that are closer to the physiological state *in vivo*. Then, we used EV-A71 at moi 100 to infect cells. At 4 h, it was observed that the virus started to induce cytopathic effects in HUVECs, which progressed rapidly and caused extensive cell death at 8 h and 12 h post-infection (Figure 1A). The cell viability of HUVECs gradually decreased (Figure 1B), EV-A71 gene (Figure 1C) and VP1 protein (Figure 1E) expression increased over time, and EV-A71 titers increased significantly at 12 h post-infection (Figure 1D). These findings suggest that HUVECs are highly sensitive to EV-A71.

Next, we examined changes of ER stress and lipid metabolism after EV-A71 infection. We used BiP protein, a marker protein of ER stress, to characterize the ER (Kopp et al., 2019). BiP protein expression increased over time (Figure 1E). This is consistent with previous findings that EV-A71 infection induces ER stress (Jheng et al., 2010). PLIN2 is a lipid droplet surface protein and is often used as a marker protein of lipid droplets (Kaushik and Cuervo, 2015). We used PLIN2 to characterize the level of lipids. PLIN2 protein were significantly decreased at 8 h and 12 h after EV-A71 infection (Figure 1E). Nile red is a lipid dye that stains polar lipids red and neutral lipids green (Schnitzler et al., 2017). We used Nile red dye staining to observe the expression changes of polar and neutral lipids (Figure 1F), and the amounts of both neutral and polar lipids decreased with time (Figure 1G). TEM (Figure 4H) showed that the number (Figure 4I) and size (Figure 4J) of lipid droplets in EV-A71 group were smaller than those in control group. The data indicated that EV-A71 infection caused an increase in lipid synthesis and a decrease in both neutral and polar lipids.

The above results showed that the virus replication level was higher and the cell CPE effect was more obvious at 12 h than at 4 h or 8 h after EV-A71 infection. EV-A71 replication leads to changes in ER stress and lipid metabolism levels, which may be conducive to virus replication.

### Lipid metabolism, not ER stress, determines EV-A71 replication

**Figure 2:**
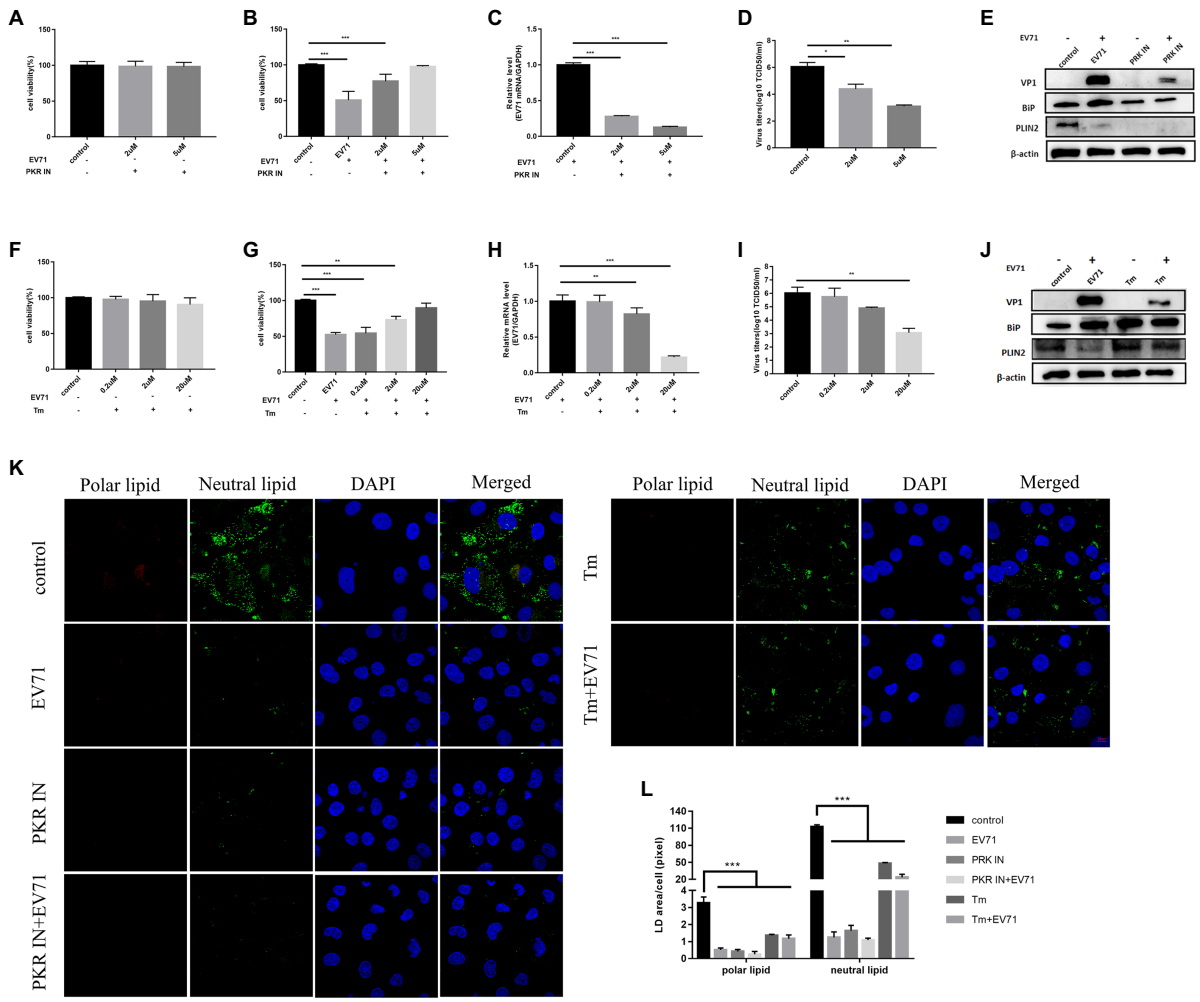
Both PKR IN and Tunicamycin inhibit the replication of EV-A71 **(A)** HUVECs were treated with 2 μM and 5 μM PKR IN for 1 h. Cell viability was detected by CCK8. **(B)-(D)** HUVECs were treated with 2 μM and 5 μM PKR IN for 1 h and then co-incubated with EV-A71 for 12 h. **(B)** the cell viability. **(C)** Total intracellular RNA was extracted and then the expression level of EV-A71 gene was detected by RT-qPCR. **(D)** collecting the culture supernatant to detect the virus titer. The virus titers were 2.24 × 10^4^TCID50/ml and 1.26 × 10^3^TCID50/ml after 2 μM and 5 μM PKR IN, respectively. **(E)** Detection of VP1, BiP and PLIN2 protein expression levels. Blank (1st channel), be infected with EV-A71 for 12 h (2nd channel), 5 μM PKR IN treated HUVECs for 1 h (3rd channel) and 5 μM PKR IN pretreated HUVECs for 1 h and then co-incubated with EV-A71 for 12 h (4th channel) VP1, BiP and PLIN2 protein expression levels. **(F)** HUVECs were treated with 0.2 μM, 2 μM and 20 μM of Tm for 12 h, then cell viability was detected by CCK8. **(G)–(I)** HUVECs were treated with 0.2 μM, 2 μM, and 20 μM Tm for 12 h and then incubated with EV-A71 for 12 h. **(G)** the cell viability. **(H)** the extraction of total intracellular RNA, the gene expression levels of EV-A71 were detected by RT-qPCR. **(I)** collecting the culture supernatant to detect the virus titer. The virus titers of the 0.2 μM, 2 μM, and 20 μM were 5.50 × 10^5^TCID50/ml, 7.76 × 10^4^TCID50/ml, and 1.16 × 10^3^TCID50/ml, respectively. **(J)** Detection of VP1, BiP, and PLIN2 protein expression levels. Blank (1st channel), be infected with EV-A71 for 12 h (2nd channel), 20 μM Tm treated HUVECs for 1 h (3rd channel) and 20 μM Tm pretreated HUVEC cells for 1 h and then co-incubated with EV-A71 for 12 h (4th channel). **(K)** Lipid droplet expression was decreased after PKR IN and Tunicamycin treatment. Nile Red staining was used to observe the expression levels of polar and neutral lipid. Control (Cell culture 12 h), EV-A71 (be infected with EV-A71 for 12 h), PKR IN (5 μM PKR IN treated for 1 h), PKR IN+EV-A71 (5 μM PKR IN pretreated for 1 h and incubated with EV-A71 for 12 h), Tm (20 μM Tm treated for 12 h), Tm + EV-A71 (20 μM of Tm pretreated for 12 h and incubated with EV-A71 for 12 h). **(L)** The lipid droplet area of each cell was quantitatively calculated. All the experiments were carried out in triplicate. Error bars represent SEM. * means *p* < 0.05, ** means *p* < 0.01, *** means *p* < 0.001.

The 12 h of EV-A71 infection was selected as a key time point for the study to facilitate understanding of the effects of ER stress and lipid metabolism. Intervening ER stress with chemical drugs PKR IN, an inhibitor of ER stress (Dankwa et al., 2021), we found that both 2 μM and 5 μM PKR IN made cell viability greater than 95% (Figure 2A). Co-incubation of PKR IN with EV-A71 made virus gene and protein (Figures 2C,E), and virus titers (Figure 2D) decreased. The cell viability increased (Figure 2B). It showed that virus replication was inhibited. BiP protein expression decreased (Figure 2E). It indicated that PKR IN could inhibit ER stress.

Next, we investigated the level of viral replication following ER stress activation by Tunicamycin (Tm). Tm is a chemical drug that can cause the accumulation of unfolded proteins in the ER and induces ER stress (Shimazawa et al., 2007). Our experiment found that the cells treated with 0.2 μM, 2 μM and 20 μM Tm kept a high-level viability (Figure 2F). BiP was significantly increased (Figure 2J). Co-incubation of Tm with EV-A71 increased the cell viability (Figure 2G) and BiP expression (Figure 2J), but interestingly decreased both EV-A71 genes (Figure 2H) and virus titers (Figure 2I). The unexpected phenomenon that Tm activated ER stress but inhibited viral replication led us to suspect that ER stress might not be the key factor affecting EV-A71 replication.

We explored lipid metabolism under the conditions of PKR IN and Tm. After PKR IN treatment, the expression levels of PLIN2 protein were all decreased (Figure 2E). However, after Tm treatment, the changes of expression levels of PLIN2 protein increased (Figure 2J). Both neutral and polar lipid were reduced after PKR IN and Tm treatment, but the reduction in PKR IN was larger than that of Tm (Figures 2K,L).

The above results suggested that ER stress has no decisive effect on EV-A71 replication, and the discovery of lipids reduction deserves further research.

### Inhibition of lipid anabolic enzymes reduces EV-A71 replication

**Figure 3:**
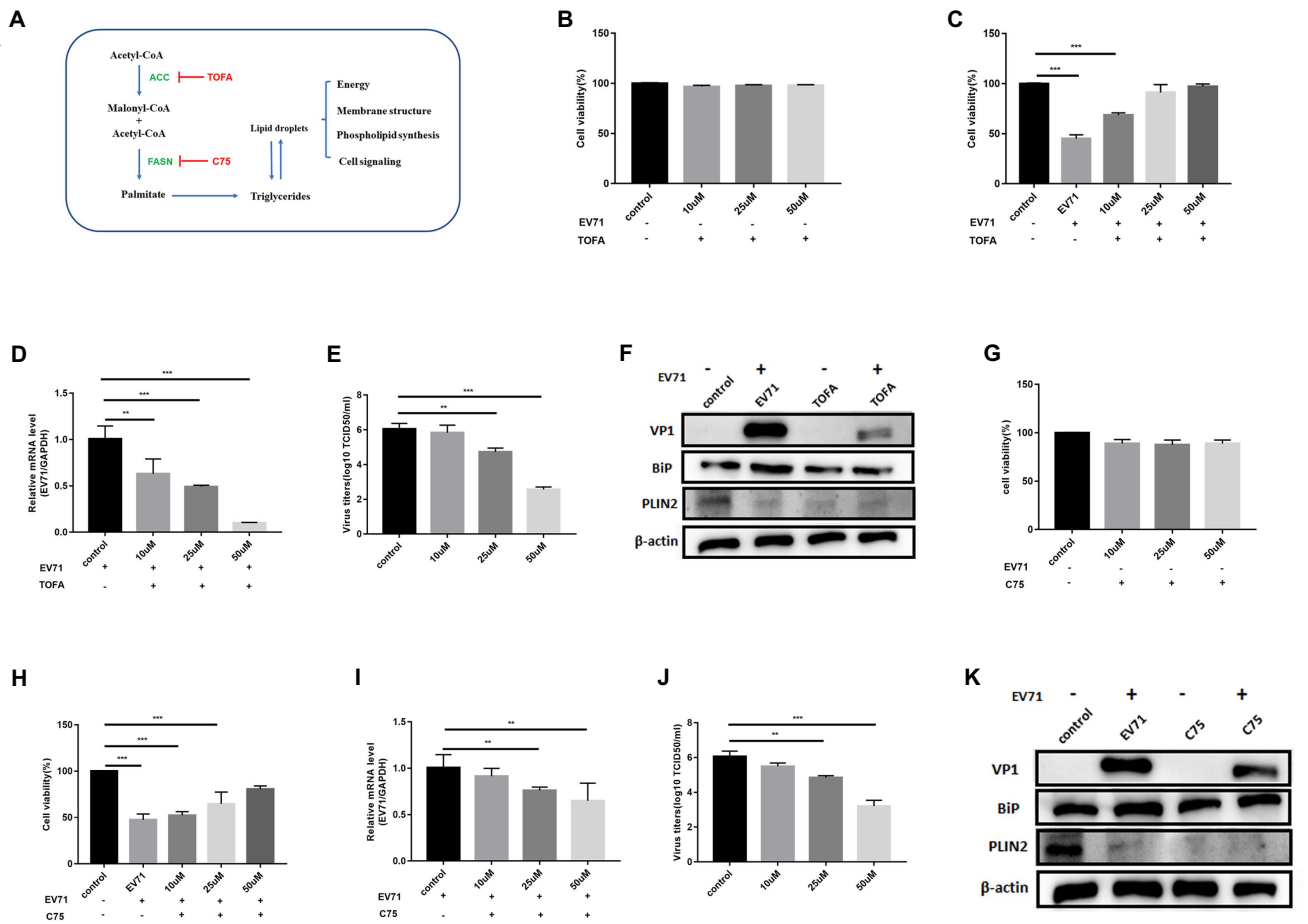
Both TOFA and C75 inhibit EV-A71 replication. **(A)** The *de novo* fatty acid biosynthetic pathway and the biological functions of lipid. Key enzymes in the pathway are highlighted acetyl-CoA carboxylase (ACC) and fatty acid synthase (FASN) as well as their cognate inhibitors TOFA and C75, respectively. **(B)** 10 μM, 25 μM and 50 μM TOFA were used to treat cells for 12 h. cell viability was detected by CCK8. **(C)–(E)** HUVECs were treated with 10 μM, 25 μM, and 50 μM TOFA for 12 h and then incubated with EV-A71 for 12 h. **(C)** Cell viability was detected with CCK8. **(D)** Total intracellular RNA was extracted and then EV-A71 gene expression levels were detected by RT-qPCR. **(E)** The culture supernatants were collected to detect virus titer. The virus titers of 10 μM, 25 μM, and 50 μM TOFA were 5.88 × 10^5^TCID50/ml, 5.12 × 10^4^TCID50/ml, 3.63 × 10^2^TCID50/ml, respectively. **(F)** Detection of VP1, BiP and PLIN2 protein expression levels. Blank (1st channel), be infected with EV-A71 for 12 h (2nd channel), 50 μM TOFA treated HUVECs for 12 h (3rd channel), and 50 μM TOFA pretreated HUVECs for 12 h and then co-incubated with EV-A71 for 12 h (4th channel). **(G)** 10 μM, 25 μM, and 50 μM of C75 were used to treat cells for 12 h. Then cell viability was detected with CCK8. **(H)–(J)** HUVECs were treated with 10 μM, 25 μM, and 50 μM C75 for 12 h then incubated with EV-A71 for 12 h. **(H)** Cell viability was detected by CCK8. **(I)** RT-qPCR detected gene of EV-A71. **(J)** The culture supernatants were collected to detect the virus titer. The virus titers of 10 μM, 25 μM, and 50 μM were 2.95 × 10^5^TCID50/ml, 6.91 × 10^4^TCID50/ml, and 1.44 × 10^3^TCID50/ml, respectively. **(K)** Detection of VP1, BiP and PLIN2 protein expression levels. Blank (1st channel), be infected with EV-A71 for 12 h (2nd channel), 50 μM C75 treated HUVECs for 12 h (3rd channel), and 50 μM C75 pretreated HUVECs for 12 h and then co-incubated with EV-A71 for 12 h (4th channel). All the experiments were carried out in triplicate. Error bars represent SEM. * means *p* < 0.05, ** means *p* < 0.01, *** means *p* < 0.001.

We next evaluated the effect of two pharmacological inhibitors on viral replication. 5-(tetradecyloxy)-2-furoic acid (TOFA) and C75, which inhibit the enzymes ACC and FASN respectively (Wong et al., 2018; Figure 3A).

**Figure 4:**
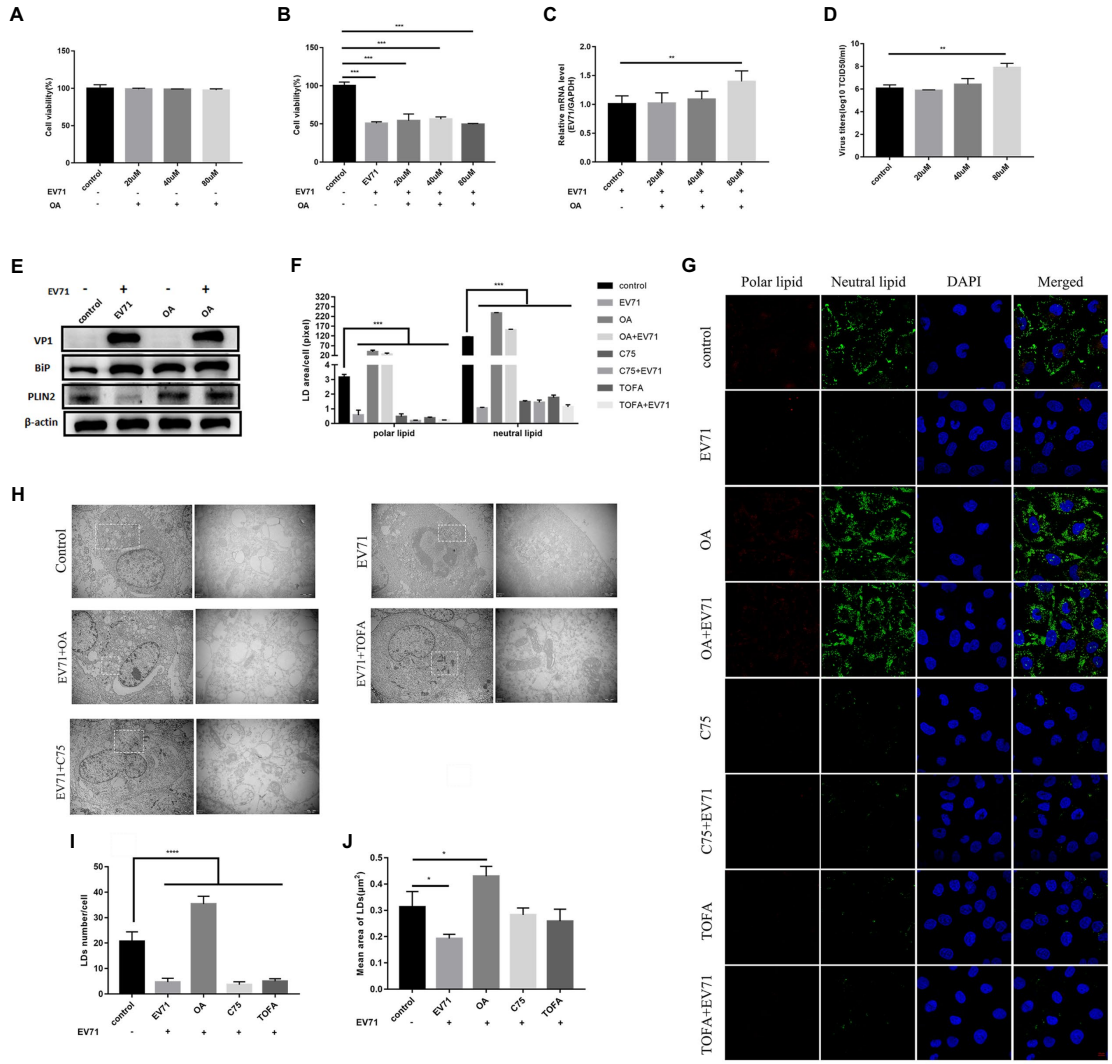
OA activates lipid metabolism and promotes EV-A71 replication. **(A)** After using20 μM, 40 μM, and 80 μM OA to treat cells for 12 h, the cell viability was detected. **(B)–(D)** HUVECs were treated with 20 μM, 40 μM, and 80 μM OA for 12 h and then incubated with EV-A71 for 12 h. **(B)** Cell viability was detected. **(C)** The expression levels of EV-A71 gene. **(D)** collecting the culture supernatant to detect the virus titer. The virus titers of the OA at 20 μM, 40 μM, and 80 μM were 7.58 × 10^5^TCID50/ml and 2.13 × 10^6^TCID50/m. **(E)** Detection of VP1, BiP, and PLIN2 protein expression levels. Blank (1st channel), be infected with EV-A71 for 12 h (2nd channel), 80 μM OA treated HUVECs for 12 h (3rd channel), and 80 μM OA pretreated HUVECs for 12 h and then co-incubated with EV-A71 for 12 h (4th channel). **(F)** Quantitative calculation of the area of fluorescence in each cell. **(G)** Lipid droplets decreased after TOFA and C75 treatment, whereas lipid droplets increased after OA treatment. Nile Red staining was used to observe the expression levels of polar and neutral lipid droplets. Control (Cell culture 12 h), EV-A71(be infected with EV-A71 for 12 h), OA (80 μM OA treated for 12 h), OA + EV-A71 (80 μM OA pretreated for 12 h and incubated with EV-A71 for 12 h), TOFA (50 μM TOFA treated for 12 h), TOFA+EV-A71 (50 μM TOFA pretreated for 12 h and then incubated with EV-A71 for 12 h), C75 (50 μM C75 treated for 12 h), C75 + EV-A71 (50 μM C75 pretreated for 12 h and incubated with EV-A71 for 12 h). **(H)** The expression levels of lipid droplet was observed by TEM. Control (Cell culture 12 h), EV-A71 (be infected with EV-A71 for 12 h), OA + EV-A71 (80 μM OA pretreatment for 12 h and then incubated with EV-A71 for 12 h), TOFA+EV-A71 (50 μM TOFA pretreated for 12 h and then incubated with EV-A71 for 12 h), C75 + EV-A71 (50 μM C75 pretreated for 12 h and incubated with EV-A71 for 12 h). **(I)** The number of lipid droplets per cell was quantitatively calculated. **(J)** The area of lipid droplets per cell was quantitatively calculated. All the experiments were carried out in triplicate. Error bars represent SEM. * means *p* < 0.05, ** means *p* < 0.01, *** means *p* < 0.001.

Cells treated with 10 μM, 25 μM, and 50 μM TOFA kept great viability (Figure 3B). The expression of PLIN2 protein (Figure 3F) and polar and neutral lipid decreased (Figure 4G), showing that TOFA can inhibit lipid metabolism. There was no significant change in BiP protein (Figure 3F). It suggested TOFA had no inhibition on ER stress. Co-incubation of TOFA with EV-A71 resulted in increasing cell viability (Figure 3C). EV-A71genes (Figure 3D),VP1 (Figure 3F) and virus titers (Figure 3E) were reduced, showing that virus replication was inhibited by TOFA. The expression of PLIN2 protein (Figure 3F) and polar and neutral lipid (Figure 4G) decreased. The number (Figure 4I) and size (Figure 4J) of lipid droplets in EV-A71 + TOFA shown by TEM (Figure 4H) were smaller than those in control. It indicated that TOFA can reduce the expression of lipid. There was no significant change in BiP protein (Figure 3F), demonstrating TOFA has no obvious effect on the ER stress.

C75 with 10 μM, 25 μM, and 50 μM made the cell viability more than 90% (Figure 3G). The expression of PLIN2 (Figure 3K) and polar and neutral lipid droplets (Figure 4G) decreased. There was no significant change in BiP (Figure 3K). It indicated that C75 could inhibit lipid metabolism but failed to stimulate ER stress. Co-incubation of C75 with EV-A71 increased cell viability (Figure 3H). EV-A71 genes (Figure 3I), VP1 (Figure 3K) and virus titers (Figure 3J) reduced. It is shown that C75 can reduce virus replication. PLIN2 protein (Figure 3K) and polar and neutral lipid (Figure 4G) decreased. TEM (Figure 4H) showed that the number (Figure 4I) and size (Figure 4J) of lipid droplets in EV-A71 + C75 were smaller than those in control. It demonstrates C75 inhibited the expression of lipid. There was no significant change in BiP protein (Figure 3K), showing that C75 has no obvious activating effect on the ER.

Our data indicate that lipid metabolism levels are closely related to EV-A71 replication, and that EV-A71 replication is also inhibited when lipid metabolism is inhibited.

### Addition of oleic acid to promote EV-A71 replication

To further explore the relationship between lipid metabolism and EV-A71 replication. We activated lipid metabolism with the activator oleic acid(OA), which is a triglyceride supplement (Mohamed et al., 2020). After treatment of cells with OA of 20 μM, 40 μM, and 80 μM, cell viability was observed to be greater than 90% (Figure 4A). The expression of PLIN2 protein (Figure 4E) and polar and neutral lipid increased (Figures 4F,G). It showed that OA activated lipid metabolism. BiP protein expressions increased (Figure 4E), suggesting ER stress was activated. After co-incubating OA with EV-A71, cell viability was at a lower level (Figure 4B). EV-A71 genes (Figure 4C), VP1 (Figure 4E), and virus titers (Figure 4D) were increased, suggesting OA increased virus replication. PLIN2 protein expressions increased (Figure 4E) and polar and neutral lipid increased (Figures 4F,G). TEM (Figure 4H) showed that the number (Figure 4I) and size (Figure 4J) of lipid droplets in EV-A71 + OA were larger than those in control. It shows OA activated lipid metabolism. BiP protein expressions increased (Figure 4E), suggesting ER stress was activated.

### β-Oxidation of lipids provides energy for EV-A71 replication

Studies have shown that lipids provide the necessary membrane structure for the replication compartment of EV-A71 (Laufman et al., 2019). Here, we synthesized previous studies and the above data. We speculate that β-oxidation of lipids provides the necessary energy production for EV-A71 replication. To directly test the hypothesis that infected cells are dependent on lipid utilization within mitochondria, cells were treated with the pharmacological inhibitor Etomoxir. Etomoxir is an irreversible inhibitor of carnitine palmitoyltransferase 1 (CPT1), which prevents the entry of long-chain fatty acids into mitochondria (Schlaepfer et al., 2014). CPT1, a key enzyme regulating fatty acid oxidation (FAO), catalyzes the conversion of acyl-CoA to acylcarnitines, which then transmembrane into mitochondria (Schlaepfer and Joshi, 2020).

**Figure 5:**
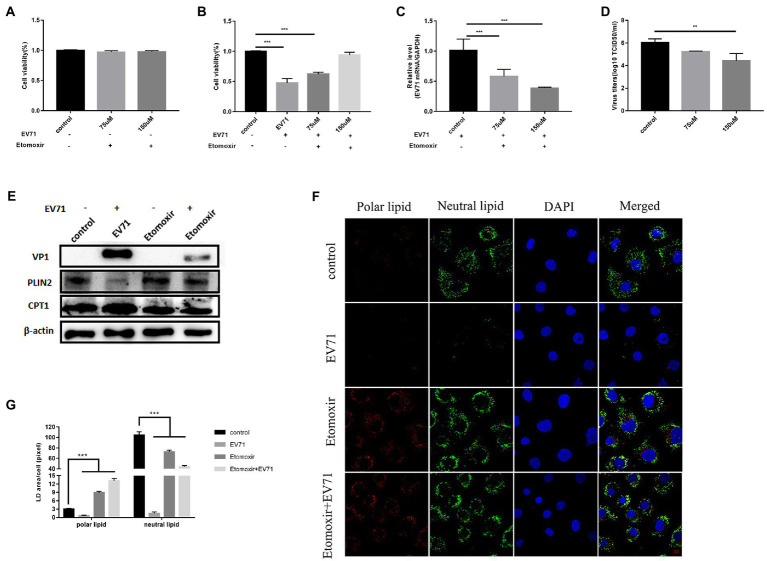
Etomoxir inhibits the replication of EV-A71. **(A)** Cells were treated with 75 μM and 150 μM Etomoxir for 12 h. cell viability was detected by CCK8. **(B)–(D)** HUVECs were treated with 75 μM and 150 μM Etomoxir for 12 h and then incubated with EV-A71 for 12 h. **(B)** The cell viability. **(C)** The expression levels of EV-A71 gene. **(D)** The culture supernatant was collected to detect virus titer. The virus titers were 1.73 × 10^5^TCID50/ml and 2.04 × 10^3^TCID50/ml at 75 μM and 150 μM. **(E)** Detection of VP1, CPT1 and PLIN2 protein expression levels. Blank (1st channel), be infected with EV-A71 for 12 h (2nd channel), 150 μM Etomoxir treated cells for 12 h (3rd channel) and 150 μM Etomoxir pretreated cells for 12 h and then co-incubated with EV-A71 for 12 h (4th channel). **(F)** The expression levels of polar and neutral lipid droplets were observed by Nile Red staining. Control (12 h), EV-A71 (be infected with EV-A71 for 12 h), Etomoxir (150 μM Etomoxir treated for 12 h), Etomoxir+EV-A71 (150 μM Etomoxir pretreated for 12 h and incubated with EV-A71 for 12 h). **(G)** The area of lipid droplets per cell was quantitatively calculated. All the experiments were carried out in triplicate. Error bars represent SEM. * means *p* < 0.05, ** means *p* < 0.01, *** means *p* < 0.001.

CPT1 expression increased after virus infection (Figure 5E). After treatment of cells with 75 μM and 150 μM Etomoxir, cell survival was higher in all cases (Figure 5A). PLIN2 protein had no significant change (Figure 5E). Expression of neutral lipid (Figure 5F) and CPT1 (Figure 5E) decreased, while polar lipid increased (Figure 5F). It shows that Etomoxir inhibited lipid β-oxidation. Co-incubation of Etomoxir with EV-A71 showed a concentration dose-dependent increase in cell survival (Figure 5B). Both EV-A71 genes (Figure 5C) and viral titers (Figure 5D) reduced. It suggests that Etomoxir inhibits EV-A71 replication. There was no significant change in PLIN2 protein (Figure 5E). CPT1 (Figure 5E) and the expression of neutral lipid decreased (Figures 5F,G), while polar lipid increased (Figure 5E). These data suggest that Etomoxir inhibits lipid β-oxidation. Lipids may be involved in EV-A71 replication through lipid β-oxidation and providing the energy required for replication.

**Figure 6:**
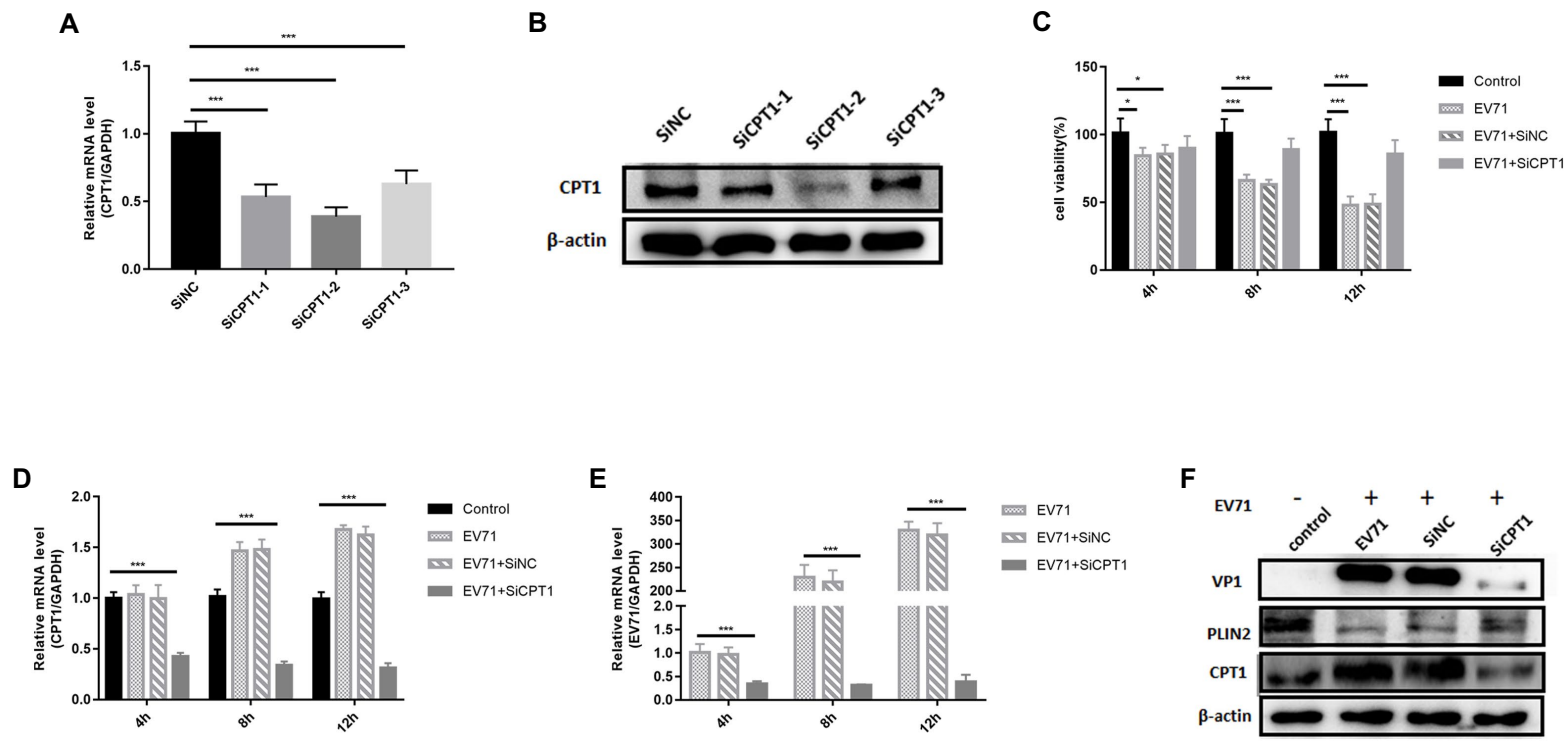
The replication of EV-A71 was inhibited after knocked down CPT1 gene. **(A,B)** SiCPT1 with the highest knockdown efficiency was screened. **(A)** The expression levels of CPT1 gene was detected by RT-qPCR. **(B)** The protein expression levels of CPT1 was detected. **(C)–(E)** 4 h, 8 h, and 12 h after virus infection. **(C)** The cell viability detected by CCK8. **(D,E)** The expression levels of CPT1 and EV-A71 gene detected by RT-qPCR. The label of control is blank, EV-A71 is be infected with EV-A71, EV-A71 + SiNC is be treated with SiNC and then be infected with EV-A71, EV-A71 + SiCPT1 is be treated with SiCPT1 and then be infected with EV-A71. **(F)** The expression levels of VP1, PLIN2 and CPT1 proteins were detected after virus infection for 12 h. the label of control is a blank, EV-A71 is be infected with EV-A71, SiNC is be treated with SiNC and then be infected with EV-A71, and SiCPT1 is be treated with SiCPT1 and then be infected with EV-A71. All the experiments were carried out in triplicate. Error bars represent SEM. * means *p* < 0.05, ** means *p* < 0.01, *** means *p* < 0.001.

To avoid the influence of non-specific targets of the drug, we knocked down CPT1 to observe the replication level of EV-A71. At the gene level, compared with the SiNC group, the knockdown efficiencies of the three siRNAs were 47% (SiCPT1-1), 62% (SiCPT1-2) and 38% (SiCPT1-3) respectively (Figure 6A). SiCPT1-2 had the highest knockdown efficiency (Figure 6B). SiCPT1-2 was selected to carry out the following studies, and the detection of CPT1 knockdown efficiency showed that compared with control, the expression of CPT1 genes and protein decreased in EV-A71 + SiCPT1 group (Figures 6D,F). The survival rate of cells was detected 4 h, 8 h, and 12 h after virus infection. Compared with control, the survival rate of EV-A71 group and EV-A71 + SiNC decreased over time, and the survival rate of EV-A71 + SiCPT1 group was no significant change (Figure 6C). After knockdown of CPT1, EV-A71 replication was reduced (Figures 6E,F). The above results show that after knockdown of CPT1, virus replication is reduced, and lipid β-oxidation can provide the necessary energy for EV-A71 replication.

## Discussion

Lipids play an integral core role in viral replication and are involved in many aspects of viral replication. After virus infection, the biosynthesis of intracellular lipid metabolism will undergo significant changes and become active, such as the formation of neutral lipids and the synthesis of long-chain fatty acids. Some viruses choose lipid metabolism pathways to generate ATP, to facilitate viral replication, or to synthesize membrane components for replication sites. DENV activates an autophagy-dependent form of lipophagy that breaks down LD into free fatty acids (FFA), which undergo β-oxidation in mitochondria to generate ATP (Heaton and Randall, 2010). Meanwhile, DENV appears to increase *de novo* production of free fatty acid (FFA) by redistributing the fatty acid synthase complex (FASN) to replication sites and upregulating its activity (Heaton et al., 2010). EV-A71 can also utilize lipids as receptors or cofactors for its entry into cells (Chazal and Gerlier, 2003; Taube et al., 2010). It can be used as a component or regulator of viral replication complexes, promoting intracellular signaling factors to regulate viral protein synthesis or the transport, assembly and release of viral particles (Nagy et al., 2016). A large number of vesicles aggregated within the host cytoplasm, known as viral replication compartments (RCs), were found in cells within hours of poliovirus (PV) infection. PV and EV-A71 proteins can link viral replication compartments (RCs) to lipid droplets (LDs), facilitating the transfer of fatty acids through lipid droplets, providing essential lipids for replication compartments (Laufman et al., 2019). After EV-A71 infection, 47 lipids within 11 lipid classes in host cells were significantly disturbed, especially arachidonic acid (AA), docosahexaenoic acid (DHA), Four polyunsaturated fatty acids (PUFAs) of docosapentaene acid (DPA) and eicosapentaenoic acid (EPA) continued to be significantly upregulated after EV-A71 infection, while exogenous supply of AA, DHA and EPA significantly reduced EV-A71 replication levels (Yan et al., 2019).

In this study, we found that lipids play an indispensable role in the replication of EV-A71, while ER stress is only a cellular stress change accompanying virus replication and has no restrictive effect on virus replication. Our data suggest that triglyceride synthesis, mitochondrial import and lipid β-oxidation work together on viral replication. A key role of triglycerides (lipid droplets) is β-oxidation within the mitochondria to generate acetyl-CoA, which in turn drives oxidative phosphorylation and ATP production. Given the robust synthesis of viral RNA, DNA and proteins and the assembly of large numbers of complex virions, it seems intuitive that EV-A71 replication requires high levels of ATP.

First, we verified that EV-A71 infection can activate ER stress and alter lipid metabolism, which is consistent with previous studies (Jheng et al., 2010). EV-A71 infection activated various pathways of unfolded proteins and caused an increase in the marker protein BiP. PKR IN inhibits the ER stress to reduced viral replication, but interestingly, the level of viral replication remained suppressed after Tm activated the ER stress. This led us to further explore the reasons behind it. Inhibition or activation of ER stress can reduce lipid metabolism, which prompted us to think that the inhibition of viral replication may be related to the inhibition of lipid metabolism.

We then used C75 and TOFA, inhibitors of two key enzymes in the fatty acid synthesis pathway of FASN and ACC, to determine the effect on EV-A71 replication. C75 and TOFA significantly inhibited EV-A71 replication, which is consistent with previous reports that TOFA and C75 reduced Marek’s disease virus titers (Boodhoo et al., 2019). In addition, virus replication was increased after treatment with fatty acid biosynthesis drug OA, which was similar to the findings that palmitic acid addition enhanced CSFV replication (Liu et al., 2021). It suggests that EV-A71 replication requires lipids. Overall, these preliminary results suggest that the replication of EV-A71 requires *de novo* involvement in fatty acid biosynthesis.

The synthesis of viral RNA, DNA, and proteins and the assembly of large numbers of complex virions require a large amount of energy (Thyrsted and Holm, 2021). One of the major pathways for fatty acids is β-oxidation in mitochondria to generate acetyl-CoA, which in turn drives tricarboxylic acid cycle (TCA), oxidative phosphorylation, and ATP production (Adeva-Andany et al., 2019). Therefore, we finally determined that the CPT1 inhibitor Etomoxir greatly inhibited the replication of EV-A71, and at the same time, the level of triglycerides (lipid droplets) showed a certain reduction. After further knockdown of the CPT1 gene, the replication level of EV-A71 was found to be reduced, which fully demonstrated the important role of lipid β-oxidation in EV-A71 replication.

**Figure 7:**
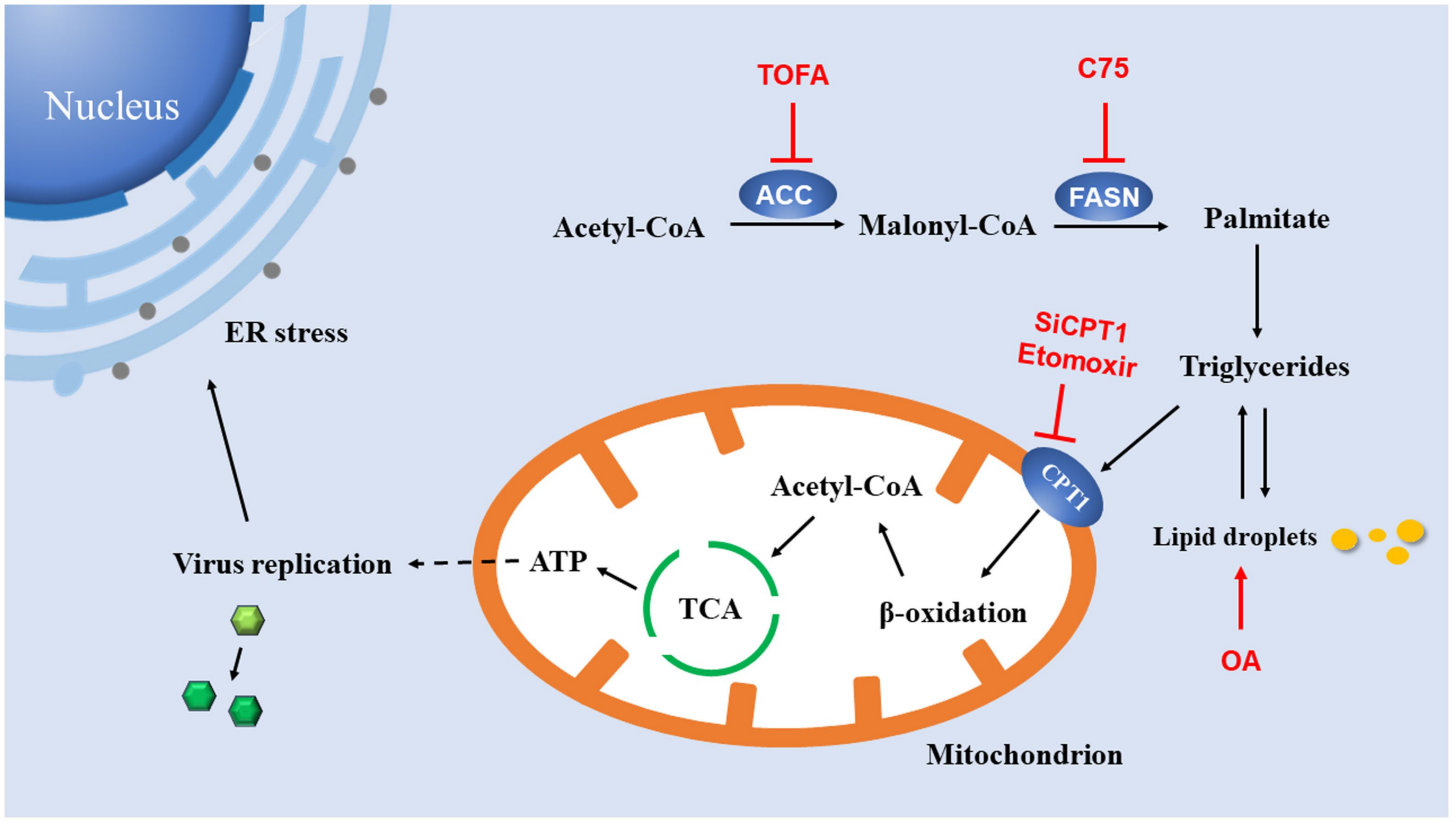
Model for lipid utilization during EV-A71 infection. The fatty acid biosynthesis pathway is used to generate ATP. Acetyl-CoA carboxylase (ACC) converts acetyl-CoA to malonyl-CoA; Fatty acid synthase (FASN) catalyzes the continuous condensation reaction of malonyl-CoA and acetyl-CoA to generate triglycerides. Triglycerides are then transported into mitochondria by carnitine palmitoyltransferase 1 (CPT1). In mitochondria, triglycerides undergo β-oxidation to acetyl-CoA, which drives the TCA cycle to generate ATP. ATP production supports DNA replication and protein synthesis of EV-A71. EV-A71 replication induces ER stress.

In summary, these data presented in this study reveal a critical role for lipids in EV-A71 replication. These data presented here support a model diagram (Figure 7). The *de novo* fatty acid biosynthetic pathway, including two enzymes ACC and FASN, generates triglycerides (lipid droplets) in the cytoplasm. Triglycerides (lipid droplets) are then imported into mitochondria *via* CPT1, where they undergo β-oxidation to produce acetyl-CoA. Acetyl-CoA drives the TCA cycle to generate a sufficient amount of energy to maximize virus production. Inhibiting any part of this pathway results in a dramatic reduction in viral replication. ATP produced by this pathway may contribute to viral replication. However, ER stress is an accompanying phenomenon in virus replication, and it has no fundamental limiting effect on virus replication. Our study also has certain limitations. We only observe the phenomenon that lipid β-oxidation was utilized by the virus to promote itself, while the specific link in the viral replication cycle that energy is mainly utilized is still worthy of further study.

## Data availability statement

The raw data supporting the conclusions of this article will be made available by the authors, without undue reservation.

## Author contributions

BZ, LZ, and XY designed the study and wrote the manuscript. BZ and WZ revised the manuscript. JYC and ZL performed the experiments and JTC processed the data. SH, SZ, ZQ YZ, JYC, JTC and GC contributed to the critical examination of the manuscript. All authors contributed to the article and approved the submitted version.

## Funding

This study was supported by grants from the National Natural Science Foundation (No. 31670168), the Guangdong Provincial Natural Science Foundation Project (No. 2018A030313767), the Guangdong Provincial Science and Technology (No. 2018B020207006), and the Guangdong Science and Technology Program key projects (No. 2021B1212030014).

## Conflict of interest

The authors declare that the research was conducted in the absence of any commercial or financial relationships that could be construed as a potential conflict of interest.

## Publisher’s note

All claims expressed in this article are solely those of the authors and do not necessarily represent those of their affiliated organizations, or those of the publisher, the editors and the reviewers. Any product that may be evaluated in this article, or claim that may be made by its manufacturer, is not guaranteed or endorsed by the publisher.
